# Born to move: a review on the impact of physical exercise on brain health and the evidence from human controlled trials

**DOI:** 10.1590/0004-282X-ANP-2020-0166

**Published:** 2021-06-23

**Authors:** Camila Vorkapic, Silvânia Leal, Heloisa Alves, Michael Douglas, André Britto, Estélio Henrique Martin Dantas

**Affiliations:** 1 Universidade Tiradentes Laboratório de Biociências Departamento de Medicina Aracaju SE Brazil Universidade Tiradentes, Departamento de Medicina, Laboratório de Biociências da Cinética Humana, Aracaju SE, Brazil.; 2 Universidade Federal do Estado do Rio de Janeiro Laboratório de Biociências da Cinética Humana Rio de Janeiro RJ Brazil Universidade Federal do Estado do Rio de Janeiro, Laboratório de Biociências da Cinética Humana, Rio de Janeiro RJ, Brazil.; 3 University of Massachusetts Department of Psychology Dartmouth MA United States University of Massachusetts, Department of Psychology, Dartmouth MA, United States.; 4 Universidade Estácio Departamento de Psicologia Aracaju SE Brazil Universidade Estácio, Departamento de Psicologia, Aracaju SE, Brazil.

**Keywords:** Physical Activity, Exercise, Brain, Mental Health, Review, Atividade Física, Exercício Físico, Cérebro, Saúde Mental, Revisão

## Abstract

**Background:**

Physical exercise has been found to impact neurophysiological and structural aspects of the human brain. However, most research has used animal models, which yields much confusion regarding the real effects of exercise on the human brain, as well as the underlying mechanisms.

**Objective:**

To present an update on the impact of physical exercise on brain health; and to review and analyze the evidence exclusively from human randomized controlled studies from the last six years.

**Methods:**

A search of the literature search was conducted using the MEDLINE (via PubMed), EMBASE, Web of Science and PsycINFO databases for all randomized controlled trials published between January 2014 and January 2020.

**Results:**

Twenty-four human controlled trials that observed the relationship between exercise and structural or neurochemical changes were reviewed.

**Conclusions:**

Even though this review found that physical exercise improves brain plasticity in humans, particularly through changes in brain-derived neurotrophic factor (BDNF), functional connectivity, basal ganglia and the hippocampus, many unanswered questions remain. Given the recent advances on this subject and its therapeutic potential for the general population, it is hoped that this review and future research correlating molecular, psychological and image data may help elucidate the mechanisms through which physical exercise improves brain health.

## RESUMO

**Introdução:**

Evidências das últimas décadas têm mostrado que o exercício físico impacta de forma significativa aspectos neurofisiológicos e estruturais do cérebro humano. No entanto, a maioria das pesquisas emprega modelos animais, o que gera confusão no que diz respeito aos efeitos reais do exercício no cérebro humano, assim como os mecanismos adjacentes.

**Objetivo:**

Apresentar uma atualização sobre o impacto do exercício no cérebro; revisar e analisar sistematicamente as evidências provenientes exclusivamente de estudos randomizados controlados em humanos, dos últimos seis anos.

**Métodos:**

Foi conduzida uma busca na literatura usando as bases de dados MEDLINE (via PubMed), EMBASE, Web of Science e PsycINFO, para todos os estudos randomizados e controlados publicados entre janeiro de 2014 e janeiro de 2020.

**Resultados:**

Foram revisados 24 estudos randomizados controlados em humanos, que observavam a relação entre exercício físico e alterações neuroquímicas e estruturais no cérebro.

**Conclusões:**

Ainda que esta revisão tenha observado que o exercício físico melhora a plasticidade cerebral em humanos, particularmente por meio de alterações no fator neurotrófico derivado do cérebro (BDNF), conectividade funcional, núcleos da base e hipocampo, muitas questões ainda precisam ser respondidas. Dados os avanços recentes nessa temática e seu potencial terapêutico para a população em geral, espera-se que este manuscrito e pesquisas futuras que correlacionem estudos moleculares e variáveis psicológicas e de imagem possam ajudar na elucidação dos mecanismos pelos quais o exercício físico melhora a saúde cerebral.

## INTRODUCTION

**Figure 1:**
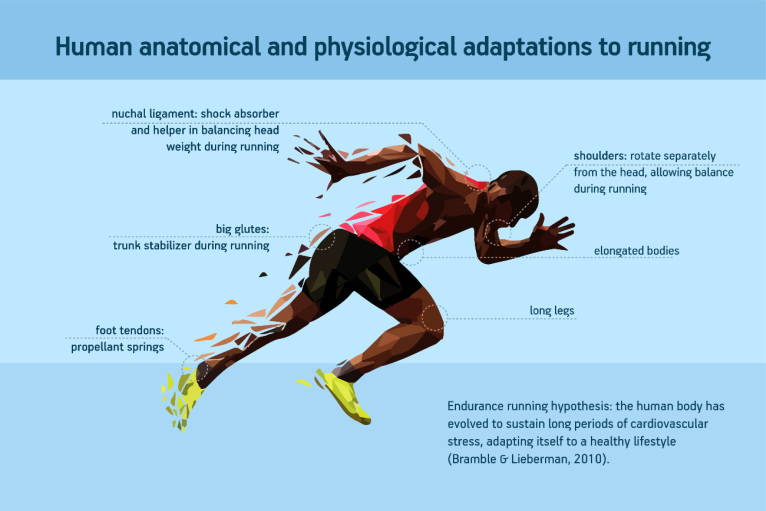
Human anatomical and physiological features that are adaptations to running, according to the endurance running theory.

In 2004, Bramble and Lieberman suggested that humans evolved from monkey-like ancestors, specifically due to their ability to run long distances. According to these authors^1^, strong selection for running was crucial in shaping the body of modern man and was an essential factor in the appearance of specific anatomical features. Figure 1 shows typical human anatomical and physiological features that are adaptations to running, according to the endurance running theory from Bramble and Lieberman^1^. The close connection between movement (exercise) and human evolution is shown by the fact that inactivity makes people physically and mentally ill^2^. Studies have shown that movement is so essential for humans that the brain not only benefits from it, but also requires it in order to function properly^3^.

The basic neurobiological mechanisms associated with physical exercise can occur at two levels: extracellular, with exercise inducing angiogenesis from pre-existing vessels; and intracellular, through increasing hippocampus neurogenesis^4^. The functional significance of this effect is still uncertain, but it has been suggested that newly formed neurons can be integrated into the existing neural network and become fully functional^5^. Exercise also seems to induce the growth of new synapses (synaptogenesis)^5^. In addition, animal studies have shown that exercise increases the synthesis of growth factors such as brain-derived neurotrophic factor (BDNF) and insulin-like growth factor (IGF-1), proteins that play a crucial role in neuroplasticity, neuroprotection and neurogenesis^4^. There is also evidence that neuromodulation and neurotransmission are regulated by physical exercise^6^^,^^7^. Lastly, an emerging concept suggests that brain health and cognitive functions are modulated by the interrelationship between central and peripheral factors^8^. Systemic inflammatory processes, which are present in metabolic diseases such as hypertension or insulin resistance, increase central nervous system inflammation and are associated with cognitive decline^8^. Human randomized controlled trials have shown that exercise upregulates neurotransmitters^9^, boosts neurotrophic factor synthesis^10^^,^^11^, increments functional connectivity^12^^,^^13^ and increases basal ganglion^14^ and hippocampus^15^^,^^16^ volume.

Studies have indicated that physical exercise reduces symptoms associated with different mental disorders, such as depression and anxiety^9^, and neurodegenerative diseases such as Alzheimer's and Parkinson's^8^. Thus, exercise forms an effective neuroprotective strategy against the deleterious effects of aging^17^^,^^18^.

Although the understanding of exercise-related molecular and cellular changes in humans is relatively limited, imaging technologies have enabled observation of changes in brain structure and function as a result of exercise in humans. Diamond^19^, for example, found that fitness training had robust but selective benefits for cognition, among which the largest benefits related to executive control processes. Other studies found that highly fit or aerobically trained participants showed better behavioral performance and greater task-related activity in the prefrontal and parietal cortices, i.e., in regions consistently implicated in attentional selection and resolution of response conflict^20^^,^^21^.

**Figure 2:**
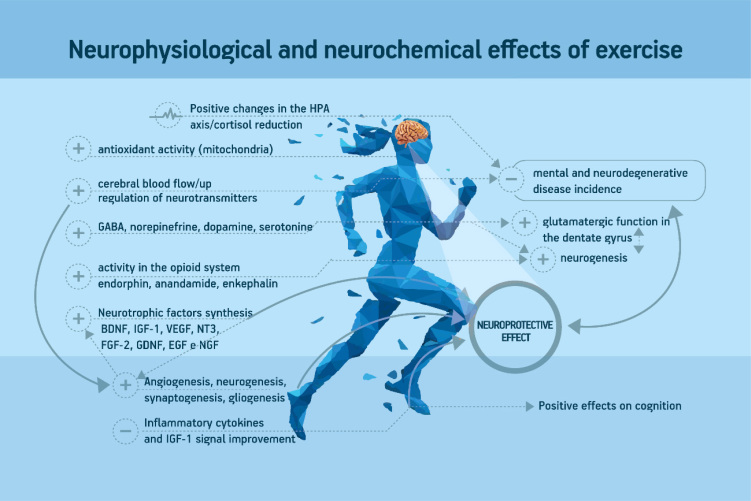
Summary of neurophysiological and neurochemical effects of physical exercise.

Neuroimaging studies have suggested that physical exercise has a protective role in preventing age-related decline and disorders, especially brain atrophy. Colcombe et al.^22^ observed significant increases in both gray matter and white matter volumes (primarily in prefrontal and temporal areas) in older adults (60–79 years), as a result of an exercise program. Erickson et al.^23^ found that aerobically trained subjects showed preservation of and increased hippocampus volume and better spatial memory performance. Erickson et al.^23^ also observed increased anterior hippocampus volume in older adults, following a long-term exercise program. Interestingly, a 1.4% decline in the control group was also observed. Other studies showed that increases in total physical activity were positively related to increases in gray matter volume in the prefrontal and cingulate cortices^24^, as well as greater white matter integrity in the frontal and temporal lobes^25^. Among the many possible mechanisms through which physical exercise yields the abovementioned improvements are the following: downregulation of the HPA axis^4^; upregulation of different neurotransmitters and neuromodulators^16^; increased neurogenesis^11^, synaptogenesis^5^ and neurotrophic factors^4^^,^^6^^,^^7^; and the interrelationship between central and peripheral factors^8^. However, at the cellular level, most of this evidence comes from animal studies^5^^,^^6^^,^^7^^,^^8^. Figure 2 summarizes the neurophysiological and neurochemical effects of exercise.

It is relevant to note that since the approach of human neuroscience is basically noninvasive, it does not allow direct measurement of exercise effects on the brain at the cellular and molecular levels. To overcome this limitation, research uses animal models^12^. However, as previously stated, this yields significant confusion regarding the real effects of exercise on human brain structures and neurochemistry, as well as regarding the underlying mechanisms involved. In addition, many human studies have methodological limitations, such as the lack of control groups or randomization. For this reason, it is crucial to elucidate the real impact of physical exercise on the human brain by examining the evidence specifically from human randomized controlled trials.

Therefore, the aims of the current article were: 1) to present an update on the impact of physical exercise on brain health; and 2) to review and analyze evidence exclusively from human randomized controlled studies from the last six years.

## METHODS

### Registration and protocol

This review was registered in the International Prospective Register of Systematic Reviews (PROSPERO) under the CRD # 4202015989. It was carried out in accordance with the Preferred Reporting Items for Systematic Reviews and Meta-Analyses (PRISMA), a 27-item checklist that includes the title, abstract, methods, results, discussion and funding*,* which is designed to help authors of systematic reviews and meta-analyses.

### Search strategy

The search of the literature was conducted independently by two reviewers (CFV and SL) using MEDLINE (via PubMed), EMBASE, Web of Science and PsycINFO databases for all randomized control trials published between January 2014 and January 2020 (last six years). Studies that examined the relationship between physical exercise, structural, and neurochemical changes were scrutinized. One strategy was to frame the search in the form of a question, while allowing clarifications needed for selecting relevant results: Does exercise or physical activity cause structural, neurochemical and neurophysiological changes in the brain of healthy adults, according exclusively to RCTs?

Another strategy used for creating a searchable question was to put it in the form of a PICO question. PICO only partially applied to our research question, but the principle of breaking the question into searchable parts is useful and has been applied:

- **P:** Population: healthy adults.
- **I:** Intervention: exercise and physical activity.
- **C:** Comparison: control.
- **O:** Outcome: structural, neurochemical and neurophysiological changes in the brain.

We searched the databases using mainly keywords and controlled vocabularies. Because of the diverse nature of the relationship between exercise and the nervous system, different keywords can be applied. So the choice of specific keyword was based on the current literature in the field of exercise neuroscience. A simple chart was set up in order to help organize the searching. A column representing each idea and two correlated rows was created: one row for the controlled vocabulary terms and the other for the synonyms and phrases that express the idea in a keyword search. The terms within the column were combined with OR, while different columns were combined with AND. Consequently, a Boolean logic using the three most common operators (*AND, OR* and *NOT*) was applied. Studies that examined the relationship between exercise or physical activity and brain changes were scrutinized. The search strategy included studies, abstracts, titles and keywords, as follows:

1. ((exercise OR physical activity OR exercise program OR exercise intervention OR physical activity intervention OR physical activity program) AND (brain OR brain changes OR brain volume OR structural changes) AND (healthy) AND (adults)) NOT children.
2. ((exercise OR physical activity OR exercise program OR exercise intervention OR physical activity intervention OR physical activity program) AND (neurochemical changes OR neurophysiological changes OR gray matter OR white matter OR connectivity OR cerebral blood flow OR hippocampus OR cortex OR prefrontal cortex OR cortical activity OR neurotransmitters OR neurotrophic factors) AND (healthy) AND (adults)) NOT children.

### Study selection criteria

Studies that investigated the relationship between physical exercise and structural or neurochemical changes were included in the systematic review. Studies were considered eligible only if: (1) they were human randomized controlled trials (RCTs); (2) they investigated healthy adults; (3) they were published or accepted for publication in a peer-reviewed journal; (4) interventions included an aerobic exercise program; (5) intervention programs included other types of physical activity, such as dance, sports and resistance training; (6) interventions included acute or chronic exercise; (7) interventions included observation of the impact of exercise on any brain structure (not function), volume, connectivity and blood flow; and (8) interventions included observation of the impact of exercise on the brain's neurochemistry (neurotransmitters, neuromodulators or neurotrophic factors).

Studies were excluded if: (1) they were not randomized controlled trials (RCTs); (2) they investigated individuals suffering from any diseases; (2) they were conducted on children or adolescents; (3) they were cross-sectional, reviews or study protocols; (4) they were animal studies; (5) the outcome variable was not the impact of physical exercise on brain structures or neurochemistry; (6) they were published in any language other than English; and (7) they were published before 2014.

### Search data extraction

Two authors (EHMD and AB) separately screened abstracts, titles, and texts of the retrieved studies. They removed duplicates and excluded those that did not meet the selection criteria. Subsequently, two other authors (MD and HV) collected the following data from each article that had been selected: (1) year of publication; (2) sample; (3) intervention characteristics; (4) variables of interest; and (6) outcomes.

### Risk of bias

After the phases of search strategy, selection criteria and data extraction, the author CFV assessed the methodological risk of bias of the studies through the Quality Assessment Tool for Quantitative Studies (QATQS), which was developed by the Effective Public Health Practice Project (EPHPP, 1998). QATQS is a tool that provides a standardized means to assess study quality and develop recommendations for study findings. This quality appraisal tool was developed as an important step within the systematic review process. The final results from using the QATQS gave rise to overall methodological ratings of strong (no weak ratings), moderate (one weak rating) or weak (two or more weak ratings) in eight sections: 1) selection bias; 2) study design; 3) confounders; 4) blinding; 5) data collection methods; 6) withdrawals and dropouts; 7) intervention integrity; and 8) analysis. Any disagreements were resolved by a third researcher (RV). Table 1 shows the assessment of study quality through the QATQS.

**Table 1:** Effective public healthcare practice quality assessment (quality assessment tool for quantitative studies).

Study	Selection bias	Study design	Confounders	Blinding	Data collection methods	Withdrawals and drop-outs	Intervention integrity	Analyses	Overall rating Strong: no weak ratings, Moderate: one weak rating, Weak: two or more weak ratings
Nagamatsu et al., 2016^34^	1	1	3	3	1	3	1	1	WEAK
Neimann et al., 2014^35^	1	1	3	3	1	3	1	1	WEAK
Best et al., 2015 ^26^	1	1	1	3	1	3	1	1	WEAK
Nocera et al., 2016^36^	1	1	3	3	1	3	1	1	WEAK
Demiracka et al., 2015^27^	1	1	2	3	1	3	1	1	WEAK
Suwabe et al., 2018^38^	1	1	2	3	1	3	1	1	WEAK
Oliveira et al., 2019^37^	1	1	2	3	1	3	1	1	WEAK
Church et al., 2016^10^	1	1	2	3	1	3	1	1	WEAK
Hriv et al., 2017^29^	1	1	2	3	1	3	1	1	WEAK
Gregoire et al., 2019^28^	1	1	2	3	1	3	1	1	WEAK
Kim J et al., 2018^30^	1	1	2	3	1	3	1	1	WEAK
Marston et al., 2019^33^	1	1	2	3	1	3	1	1	WEAK
Vaughan et al., 2014^11^	1	1	2	1	1	3	1	1	WEAK
Forti et al., 2015^6^	1	1	2	1	1	3	1	1	WEAK
Magon et al., 2016^32^	1	1	3	3	1	1	1	1	WEAK
Maddock et al., 2016^9^	1	1	2	3	1	1	1	1	MODERATE
Matura et al., 2017^31^	1	1	3	1	1	3	1	1	WEAK
Zschucke et al., 2014^41^	1	1	3	3	1	3	1	1	WEAK
Tamura et al., 2014^39^	1	1	1	3	1	3	1	1	WEAK
Wagner et al., 2017^12^	1	1	2	1	1	3	1	1	WEAK
Varma et al., 2015^14^	1	1	2	1	1	1	1	1	MODERATE
Rosano et al., 2017^40^	1	1	2	3	1	1	1	1	MODERATE
Kleemeyer et al., 2015^13^	1	1	2	3	1	1	1	1	MODERATE
Kim L et al., 2017^17^	1	1	2	3	1	1	1	1	MODERATE

## RESULTS

**Figure 3:**
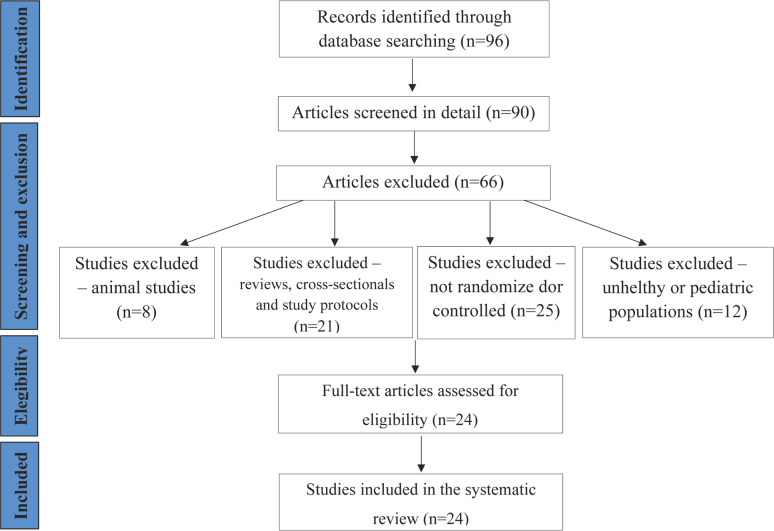
PRISMA diagram showing the study extraction flow.

The search yielded a total of 96 potentially eligible articles: 52 from Medline, 14 from Embase, 24 from Web of Science and 6 from PsycINFO. After removing 6 duplicates, 90 were screened in detail. A total of 66 studies were excluded from the review because: a) they were animal studies (8); b) they were reviews, meta-analysis, study protocols or cross-sectional studies (21); c) they were not randomized or controlled (25); and d) they were conducted on non-healthy or pediatric populations (12). In the end, a total of 24 studies met the inclusion criteria (S1^26^, S2^10^, S3^27^, S4^6^, S5^28^, S6^29^, S7^17^, S8^30^, S9^13^, S10^9^, S11^31^, S12^32^, S13^33^, S14^34^, S15^35^, S16^36^, S17^37^, S18^38^, S19^39^, S20^40^, S21^14^, S22^11^, S23^12^ and S24^41^). These were assessed for eligibility and later included in this review. The study extraction flow is demonstrated in the PRISMA diagram (Figure 3).

The studies included were published between 2014 and 2020 and were all randomized controlled trials, with sample sizes ranging from 20 to 155 subjects, aged between 21 and 65 years. The main interventions used in these studies were aerobic exercise^15^, resistance training^6^, coordination exercises^3^ or a combination of coordination and cognitive exercises^1^, calisthenics^1^ or a mixed program^3^.

The risks of bias of the studies included are displayed in Table 1. The risk of performance bias was high in these studies because it was difficult to blind participants or exercise coaches, but six studies reported blinding of the outcome assessors (S4, S11, S18, S21, S22 and S23). The risk of attrition bias was high in most studies (due to unreported data), except for six (S7, S9, S10, S12, S20 and S21) Nine studies used an active control group (S1, S2, S4, S5, S9, S14, S15, S16 and S24), instead of a no-intervention control and four studies did not inform the type of control used (S8, S19, S21 and S23).

The total duration of the interventions ranged from one session (acute) (S10, S18 and S24) to 6 to 52 weeks (S1, S2, S3, S4, S5, S6, S7, S8, S9, S11, S12, S13, S14, S15, S16, S17, S19, S20, S21, S22 and S23) and to two years (S19 and S20).

The total number of minutes (volume) spent on the interventions ranged from 10 to 30 minutes during acute protocols (S10, S18 and S24) to approximately 40 to 180 minutes/week (S1, S2, S3, S4, S5, S6, S7, S8, S11, S12, S13, S14, S15, S16, S17, S19, S20, S21, S22 and S23). The overall frequency ranged from 1 to 7 times per week.

Some studies provided data regarding changes in neurotransmitters (S10, S17 and S23) or neurotrophic synthesis (S2, S4, S5, S6, S8, S11, S13, S22 and S23). In these studies, positive correlations were observed between exercise and increased neurotrophic factors (S2, S4, S5, S8, S13, S22 and S23) and between exercise and upregulation of neurotransmitters (S10). Four studies found that exercise did not increase the levels of neurotrophic factors (S6, S11 and S17) or neurotransmitters (S17). Studies also observed increases (S18 and S23) or no differences in functional connectivity (S12) or in gray matter volume (S11). One study found that exercise increased white matter volume (S19) or reduced white matter atrophy (S1). Brain activity in the hippocampus area was found to increase after exercise (S24) or decrease but correlate with better cognition (S16). With regard to structural changes, seven studies investigated the effects of exercise on basal ganglion volume (S14 and S15) and on hippocampus volume (S7, S9, S20, S21 and S23). One study (S14) found no differences between groups regarding basal ganglion volume, but that individuals with declines in mobility levels had significant decreases in left putamen volume. Another study found that motor fitness, but not cardiovascular fitness, was positively related to the volume of the putamen and the globus pallidus (S15). All studies that investigated changes in the hippocampus found a positive correlation between exercise and increased hippocampus volume (S7, S9, S20, S21 and S23). Table 2 summarizes the information in the studies.

**Table 2:** Summary of the characteristics of the randomized controlled trials.

Study	Sample	Intervention	Control condition	Intensity of intervention	Volume and frequency	Duration	Variables of interest	Outcomes
Best et al., 2015 (S1)^26^	n=155	Resistance training	Balance and toning exercises	High	60 min 3x/week	52 weeks	Brain volume, mood and cognition.	1. Resistance training improves memory, reduces cortical white matter atrophy and increases peak muscle power executive function, compared with balance-and-toning. 2. These effects persisted at 2-year follow-up, relative to balance-and-toning.
Church et al., 2016 (S2)^10^	n=20	High-intensity low-volume (HI)	Low-intensity high-volume (HV)	Moderate to high	Duration not informed 4x/week	8 weeks	Plasma BDNF	1. BDNF response is significantly elevated after both high-intensity and high-volume training protocols.
Demirakca et al., 2016 (S3)^37^	n=21	Coordination exercises + cognitive training	Rest	n/a	60 min, 1x/week	13 weeks	Functional connectivity (rs-fMRI)	1. Significant connectivity alterations occurred between the visual cortex and parts of the superior parietal area (BA7). Premotor area and cingulate gyrus were also affected.
Forti et al., 2015 (S4^6^	n=56	High and low resistance training	Mixed low resistance training	Low and high	Duration not informed 3x/week	12 weeks	Plasma BDNF	1. Only the mixed-low-resistance training program (high number of repetitions at a sufficiently high external resistance) was able to increase circulating BDNF in older male participants. 2. Training to volitional fatigue might be necessary to obtain optimal results.
Gregoire et al., 2019 (S5)^28^	n=34	Lower body strength + aerobic training (LBS-A) and upper body strength + aerobic training (UBS-A)	Gross motor activities (GMA).	Not informed	60 min, 3x/week	8 weeks	Plasma BDNF and cognition	1. All interventions resulted in improved cognitive functions but the GMA intervention induced a larger increase in plasma BDNF concentration (cognition improvement could occur without concomitant detectable changes in BDNF). 2. No correlation was observed between changes in BDNF concentrations and cognitive performances.
Hvid et al., 2017 (S6)^29^	n=47	Progressive high-intensity power training	No intervention	Moderate to high	Approx. 45 min, 2x/week	12 weeks	Serum BDNF (mature and total)	1. Baseline systemic levels of serum mBDNF and tBDNF were not affected by exercise training.
Kim L et al., 2017 (S7)^17^	n=21	Strength training	No intervention	Moderate	50-60 min, 3x/week	24 weeks	Hippocampus volume	1. Hippocampus volume was significantly increased in the strength exercise group, but decreased in the control group.
Kim J et al., 2018 (S8)^30^	n=26	Aquarobic exercise program	Not informed	Moderate	60 min, 2x/week	12 weeks	Plasma BDNF and irisin	1. Significantly higher serum irisin and BDNF levels in the exercise group than in the control group were found. 2. Serum irisin and BDNF levels were significantly higher 30 min after the first exercise session and 30 min after the last exercise session.
Kleemeyer et al., 2015 (S9)^13^	n=52	High-intensity aerobic exercise	Low-intensity aerobic exercise	Low-vigorous	25-55 min, 2-3x/week	6 months	Hippocampus volume and microstructure	1. More positive changes in fitness were associated with more positive changes in tissue density and more positive changes in tissue density were associated with more positive changes in volume. 2. Fitness-related changes in hippocampus volume may be brought about by changes in tissue density.
Maddock et al., 2016 (S10)^9^	n=38	Aerobic exercise	Rest	Vigorous	30 min	Acute	Cortical glutamate and GABA levels (MRS)	1. Results showed that glutamate and GABA signals increased significantly in the visual cortex following exercise. In addition, there was an increase in glutamate following exercise in the anterior cingulate cortex. 2. The results are consistent with an exercise-induced expansion of the cortical pools of glutamate and GABA.
Matura et al., 2017 (S11)^31^	n=53	Aerobic exercise	Waiting list	Moderate	30 min, 3x/week	12 weeks	Brain metabolism, gray matter (GM) volume and cognition.	1. Cerebral choline concentrations remained stable in the exercise group, while increasing in the control group. 2. No effect of training was seen on cerebral N-acetyl aspartate or BDNF levels and no changes in cortical GM volume in response to aerobic exercise. 3. Stable choline concentrations in the intervention group might indicate a neuroprotective effect of aerobic exercise.
Magon et al., 2016 (S12)^32^	n=28	Slackline training	Educational Sessions	n/a	90 min, 3x/week	6 weeks	Functional connectivity (MRI)	1. MRI analysis did not reveal morphological or functional connectivity differences before or after the training between the intervention and control groups. 2. However, subsequent analysis in subjects with improved slackline performance showed a decrease of connectivity between the striatum and other brain areas during the training period, which means an increased efficiency of the striatal network.
Marston et al., 2019 (S13)^33^	n=45	High-load resistance training and moderate-load resistance training	No intervention	Moderate to high	Duration not informed 2x/week	12 weeks	Peripheral growth factors and homocysteine	1. High-load or moderate-load resistance training twice per week for 12 weeks has no effect on peripheral growth factors or homocysteine in healthy late middle-aged adults.
Nagamatsu et al., 2016 (S14)^34^	n=101	Aerobic exercise	Toning exercises	Moderate	40 min, 1x/week	12 months	Mobility and basal ganglion volume	1. In both groups, no differences were observed in the putamen volume regardless of change in mobility. 2. However, those who declined in mobility levels significantly decreased in left putamen volume.
Neimann et al., 2014 (S15)^35^	n=92	Aerobic exercise and coordination training	Stretching and relaxation	Moderate	45-60 min, 3x/week	12 months	Basa ganglion volume	1. Motor fitness but not cardiovascular fitness was positively related with the volume of the putamen and the globus pallidus. 2. Coordination training increased caudate and globus pallidus volume.
Nocera et al., 2017 (S16)^36^	n=32	Aerobic exercise	Balance exercises	Moderate to vigorous	20-45 min, 3x/week	12 weeks	Brain activity during cognitive tasks	1. Cognition (verbal fluency) was improved in the aerobic exercise group, compared with controls. 2. fMRI comparisons of IFG (inferior frontal gyrus) activity showed lower activity in the right IFG following the intervention in the aerobic group, compared with controls.
Oliveira et al., 2019 (S17)^37^	n=34	Aerobic exercise	Waiting list	Moderate	40 min, 3x/week	12 weeks	Plasma anandamide (AEA), mood and body weight	1. Regular moderate aerobic exercise reduces plasma AEA levels. 2. This reduction was associated with weight loss and improved mood, in particular, reduced anger.
Suwabe et al., 2018 (S18)^38^	n=36	Aerobic exercise	Rest	Low	10 min	Acute	Functional connectivity during cognitive task	1. A single 10-min bout of exercise increased functional connectivity between hippocampus DG/CA3 and cortical regions. 2. The magnitude of the enhanced functional connectivity predicted the extent of memory improvement.
Tamura et al., 2014 (S19)^39^	n=110	Calisthenics	Not informed	Moderate	10 min/day, everyday	2 years	Brain volume and cognition	1. The exercise group showed significant improvements in attentional shift. 2. Neuroimaging analysis revealed the significant preservation of bilateral prefrontal volume in the exercise group. 3. The longitudinal changes in attentional shift and memory were positively correlated with the prefrontal volumetric changes.
Rosano et al., 2017 (S20)^40^	n=27	Aerobic exercise	Health education	Moderate	Not reported	2 years	Hippocampus volume	1. Increased volume of the left hippocampus, left cornu ammonis and right hippocampus in the intervention group.
Varma et al 2015 (S21)^14^	n=92	Aerobic exercise (walking)	Not informed	Low to vigorous	10,000 steps/day threshold (pedometer)	Not informed	Hippocampus volume	1. A greater amount, duration, and frequency of total daily walking activity were each associated with larger hippocampus volume among older women, but not men. 2. These relationships were specific to hippocampus volume, compared to the thalamus, used as a control brain region, and remained significant for low-intensity walking activity, independent of moderate to vigorous-intensity activity and self-reported exercise
Vaughan et al., 2014 (S22)^11^	n=49	Multimodal exercise program (cardiovascular, strength and motor fitness)	Waiting list	Not reported	60 min, 2x/week	16 weeks	BDNF and cognition	1. The exercise program resulted in neurocognitive and physical performance improvements and increased levels of plasma BDNF, in older women, compared with controls. 2. Increases in BDNF levels imply neurogenesis may be a component of the mechanism underpinning the cognitive improvements associated with exercise.
Wagner et al., 2015 (S23)^12^	n=34	Aerobic exercise	Not informed	Moderate	60 min, 3x/week	6 weeks	Hippocampus volume, hippocampus glutamate/ glutamine and NAA (N-acetyl aspartate).	1. A positive correlation between the degree of fitness improvement and increased BDNF levels was found. 2. A volume decrease of about 2% of the hippocampus was negatively correlated with fitness improvement and increased BDNF levels; and positively correlated with increased TNF-α concentrations. 3. A decrease in glutamate-glutamine levels was observed in the right anterior hippocampus in the exercise group only.
Zschucke et al., 2014 (S24)^41^	n=40	Aerobic exercise	Light stretching exercises	Moderate	30 min	Acute	Brain activation (fMRI) during stress task (MIST) cortisol and amylase	1. Participants of the aerobic group showed a significantly reduced cortisol response to the MIST, which was inversely related to the previous exercise-induced amylase and cortisol fluctuations. 2. Higher bilateral hippocampus activity and lower prefrontal cortex (PFC) activity was observed in the aerobic group.

## DISCUSSION

The aim in this study was to review data exclusively from human randomized controlled studies conducted among healthy adults. The review systematically examined the literature from the last six years with regard to the effects of physical exercise on brain volume, structures, functional connectivity and neurochemical factors such as neurotransmitters, BDNF and the HPA axis, control groups or randomization.

Among the studies that observed changes in neurotrophic factors (S2, S4, S5, S6, S8, S11, S13, S22 and S23), all of them used exercise programs that lasted more than six weeks (regular exercise). Seven studies found a positive correlation between exercise and increased plasma or serum BDNF (S2, S4, S5, S8, S13, S22 and S23). These results are in agreement with an extensive meta-analysis conducted by Szuhany et al.^42^, which demonstrated the strength of the association between exercise and increased BDNF levels in humans. The review showed a moderate effect size for increases in BDNF after acute exercise. In addition, the effect of an exercise session on BDNF levels was intensified by regular exercise. These authors explained that each episode of exercise results in a “dose” of BDNF activity and that the magnitude of this “dose” can be enhanced over time by regular exercise.

In the present review, most studies that found a correlation between physical exercise and increased plasma BDNF (pBDNF), used moderate to high-intensity exercise as part of the main intervention. Unfortunately, several other studies did not report the intensity level used. In the literature, other studies have also found that exercise-induced BDNF effects in humans follow a dose-dependent relationship with regard to duration and intensity of exercise, such that the best outcomes are linked to moderate exercise^43^. A recent review by Knaepen et al.^44^ found that high intensities and graded exercise tests elicited the greatest exercise-induced increases in pBDNF concentration in healthy participants. In acute protocols, this increase has been shown to last post-exercise, to some extent. Another interesting fact is that many of the studies reviewed here not only used aerobic exercise, but also used multimodal protocols, resistance training and coordinative exercises (S2, S4, S5 and S22). Accordingly, there is evidence that increases in pBDNF concentrations can be observed in response to a variety of exercise protocols and types^45^^,^^46^. Rasmussen et al.^47^ and Tang et al.^48^ also observed effects that indicate that an acute exercise-induced increase in pBDNF is stable in response to different exercise types and protocols. According to the evidence reviewed here, moderate-intensity multimodal exercises are more effective in promoting increases in peripheral levels of BDNF, although it is still not possible to draw definite conclusions or to establish recommendation protocols for the type and intensity of exercises in a multimodal program that would be required in order to produce an increase in BDNF levels. Vedovelli et al.^49^, observed that a combined intervention for increased muscle strength and aerobic conditioning can increase BDNF levels, and that aerobic conditioning is at least partially responsible for that improvement. These authors also stated that BDNF could be a key component of the beneficial effects of physical activity on cognitive functioning, since this neurotrophin can modulate neurogenesis, neuroplasticity and neuronal survival. In addition, Vaughan et al.^11^, observed that human studies had found that motor fitness (balance, flexibility, co-ordination, agility and reaction time ability) was associated with brain activation patterns that differed from those related to cardiovascular fitness. Motor fitness training entails complexity that requires sustained attention and concentration, thereby increasing the cognitive load and evoking positive neuroplasticity. Although promising, a greater number of studies, with larger samples and less methodological biases, are needed in order to better elucidate the relationship between BDNF and multimodal exercise.

In the present review, one study (S17) observed a negative correlation between moderate regular exercise and decreased peripheral anandamide levels (AEA). It was also observed that this reduction was associated with weight loss and improved mood. Other data corroborate these findings. In a study by Matias et al.^50^, salivary AEA levels were positively correlated with body mass index, waist circumference and fasting insulin levels. Preclinical studies have also indicated that AEA has a negative effect on peripheral metabolism by impairing insulin signaling and mitochondrial function^51^. Although acute aerobic exercise has been shown to increase circulating AEA^50^, Gasperi et al.^52^ found increased upregulation and activity of resting fatty acid amide hydrolase (FAAH) — a major enzyme responsible for AEA breakdown — in the lymphocytes of physically active young men, compared with sedentary young men. The observed lower circulating AEA levels associated with improved mood, however, seems to contradict the current understanding of the relationship between exercise-related mood enhancement and endocannabinoids^53^^,^^54^. Several studies have indicated that endocannabinoids have stress-buffering, anxiolytic and antidepressant effects via CB1 receptors^55^. On the other hand, Antunes et al.^56^ showed that reduced resting plasma AEA in exercise-addicted runners was accompanied by higher negative mood scores. Such discrepancies might be due to the distinct effects of acute versus chronic exercise, measures used during exercise versus resting conditions and heterogeneity among the samples. Endocannabinoid responses to acute and chronic exercise among healthy people deserve further investigation.

In this review, two studies observed the effects of acute and regular physical exercise on other neurotransmitters. One study (S10) found that acute exercise increases the levels of GABA and glutamate in the anterior cingulate and visual cortices. During acute aerobic exercise, in the process of aerobic glycolysis, glucose is broken down to pyruvate, which then further breaks down to lactate or lactic acid. When exercise transitions from an aerobic to an anaerobic nature, the “anaerobic threshold” is met. Beyond this point, lactic acidosis occurs. Lactate is able to cross the blood-brain barrier, but is independently made by astrocytes in the brain, where it serves as a precursor of glutamate. Glutamate is then taken up by astrocytes and converted to glutamine in the glutamate-glutamine cycle^57^. Two recent studies used proton magnetic resonance spectroscopy (H1MRS) to investigate brain-level changes in lactate, glutamate and glutamine^58^ and revealed that lactate, glutamate and glutamine levels transiently increased by approximately 20% in the human cortex. Acute exercise has been known to increase peripheral lactate levels and, even though direct quantification of acute exercise-induced brain lactate levels in humans is difficult, these results were also observed in S10.

Another study analyzed in this review (S23) provided interesting results: a decrease in glutamate-glutamine levels in the right anterior hippocampus in the exercise group that seemed to be correlated with a volume decrease in the hippocampus of about 2%. The authors of that study stated that the observed volume changes were not a consequence of a neuronal loss in the right hippocampus, but rather, resulted from potential changes in gliogenesis and/or fiber organization. Astroglia are actively involved in the uptake, metabolism and recycling of glutamate, and the glutamate-glutamine cycle between neurons and glia is a major metabolic pathway that reflects the synaptic release of glutamate. Therefore, changes in glutamate metabolism might be linked indirectly to the observed structural changes, in particular those of glial morphology^59^. Further investigations regarding changes in peripheral and central neurotransmitter levels after exercise in humans are necessary to better elucidate related mechanisms.

Two studies in this review observed increases in functional connectivity as a consequence of acute aerobic (S18) and long-term coordinative exercise (S3). One long-term study found that resistance exercise reduced white matter atrophy (S1) and a 12-week study found no differences in gray matter volume in the aerobic exercise group, compared with the control (S11). Most of these results were congruent with other studies in the literature that showed alterations in white matter and connectivity as a result of exercise. Colcombe et al.^60^ reported an anterior cluster of increased white matter after six months of exercise, in a group of elderly adults. Indeed, investigators have been witnessing significant advancements in the ability to study the connectivity between brain areas embodied by white matter (see Smith et al.^61^ for review). One recent study^62^ found a correlation between white matter integrity and changes in VO_2_ max scores in frontal and temporal white matter tracts. Interestingly, the change in white matter integrity for the aerobic training group did not significantly differ from that of a control group that participated in one year of non-aerobic exercise, thus suggesting that aspects other than aerobic exercise contributed to the observed change. Voss et al.^63^ also observed that differences in resting functional connectivity were associated with fitness level. The S3 study of this review specifically observed increased connectivity in brain areas associated with the default mode network (DMN), such as the anterior cingulate cortex and the prefrontal cortex, in the intervention group. These brain regions show a decrease in activity when external processing demands are increased. Voss et al.^63^ demonstrated that some of the functional connections within the DMN exhibit a positive correlation with VO_2_ max score and spatial memory.

Even though these are promising results, it remains necessary for future research to test whether there is specificity in exercise training-induced plasticity of brain networks.

With regard to structural changes, all the studies found significant changes in the volume of the basal ganglia and the hippocampus. Three studies found that moderate to vigorous aerobic activity was associated with a greater increase in hippocampus volume (S9, S20 and S21). These results are in agreement with those from other studies in the current literature that correlated exercise with structural changes in hippocampus volume and vasculature^64^. Erikson et al.^65^ showed that subjects with higher VO_2_ max scores had larger hippocampus volumes than those with lower VO_2_ max scores. Erickson et al.^66^ showed a correlation between the volume of the hippocampus and cardiovascular fitness in older adults. A follow-up study^64^ demonstrated that long-term aerobic exercise increased the volume of the hippocampus by 2% in elderly adults, while controls who underwent one year of stretching exercises exhibited a 1.4% decrease in hippocampus volume. Similarly, Pajonk et al.^67^ reported that there was a 12–16% increase in hippocampus size in a small group of exercising schizophrenic patients, as well as in matched controls.

Interestingly, one study in this review that used strength training also observed greater hippocampus volume (S7). The effects of resistance training on other neuroplastic factors, such as neurogenesis or BDNF level, are not clear yet^68^^,^^69^, but these results suggest that the improved communication between muscle fibers and the brain, as a result of strength training, may serve a protective role in slowing down age-related declines in hippocampus volume^70^. However, further studies are needed to confirm the mechanism of variations in hippocampus volume according to the type of exercise.

With regard to basal ganglia, one study (S14) found no differences in putamen volume in the intervention group, after 12 weeks of aerobic exercise. However, the authors of that study observed that individuals with significant declines in mobility levels also showed decreases in left putamen volume. Another study (S15) showed increased caudate and globus pallidus volume in subjects who underwent a coordinative training. Indeed, better motor fitness goes together with more frequent execution of motor-demanding exercise, thus resulting in more frequent stimulation of the corresponding sensorimotor (dorsal) part of the striatum (dorsal putamen) and output structure (globus pallidus) of the basal ganglion nuclei. Coordination training, which constantly requires adapting to new tasks, can be very similar to the early stages of motor learning, and is consequently associated with improvements in performance and activation of the striatum^71^^,^^72^. Therefore, it can be assumed that the observed volume increase found in the basal ganglia among subjects who attended the coordination training resulted in experience-dependent plasticity^73^. Another study in the literature found an association between cardiovascular fitness and caudate volume^74^ but, based on the functions of the basal ganglia, it seems reasonable to assume that the association between motor fitness and basal ganglion volume might be higher than the one between cardiovascular fitness and basal ganglion volume. Much research is still needed in order to elucidate this association. Different tools for statistical analyses, basal ganglion volume determinations, numbers of samples and intervention characteristics need to be taken into consideration.

In conclusion, studying the effects of physical exercise on brain structure and neurochemistry is still recent. While robust animal research protocols have demonstrated that aerobic exercise is a powerful modulator of structural brain plasticity, human trials have primarily focused on neuroimaging and cognitive studies, and have yielded conflicting results. The lack of methodological accuracy and the use of different types of exercise, frequency, intensity and duration hinders the meaning of results. Even though this short review found that exercise improves brain plasticity in humans, particularly through changes in BDNF, functional connectivity, basal ganglia and the hippocampus, many unanswered questions remain. Therefore, future studies in humans are needed in order to demonstrate the full potential of physical exercise (or movement in general) among healthy individuals and as a therapeutic strategy to remediate a variety of mental and neurological diseases or to lessen the burden of cognitive decline associated with aging. It is hoped that future studies correlating basic research with psychological variables and imaging studies may better elucidate the mechanisms through which physical exercise improves brain health in humans.
